# Editorial: Ubiquitin and ubiquitin-like modifications in viral infection and innate immunity

**DOI:** 10.3389/fimmu.2023.1148296

**Published:** 2023-02-02

**Authors:** Konstantin M. J. Sparrer, Éric Bergeron, Soham Gupta

**Affiliations:** ^1^ Institute of Molecular Virology, Ulm University Medical Center, Ulm, Germany; ^2^ Viral Special Pathogens Branch, Division of High-Consequence Pathogens and Pathology, Centers for Disease Control and Prevention, Atlanta, GA, United States; ^3^ Department of Pharmaceutical and Biomedical Sciences, College of Pharmacy, University of Georgia, Athens, GA, United States; ^4^ Division of Clinical Microbiology, Department of Laboratory Medicine, Karolinska Institutet, Stockholm, Sweden

**Keywords:** ubiquitin, ISG15, Nedd8, ubiquitin-like modifications, E3-ubiquitin ligase, deubiquitinases, innate immunity, viral mimicry

The Research Topic ‘*ubiquitin and ubiquitin-like modifications in viral infection and innate immunity*’ aimed to advance our knowledge and understanding of the regulation of innate immune signaling by ubiquitin and ubiquitin-like modifiers during infection, its interplay with viral proteins and the application of modern techniques to decode these interactions. Six original research papers, three mini-reviews, one review article, and one perspective article that covered various aspects of the Research Topic were eventually part of the collection.

Innate immune responses are our first line of defense against invading pathogens. Multiple signaling pathways work in concert to eliminate the invading pathogen (1). However, to mediate a rapid but balanced defensive response, the innate immune system itself has to be intricately regulated. The fate and function of the individual key signalling factors is often regulated by post-translational modifications (PTMs) (2). Most prominently among them are ubiquitin (Ub) and ubiquitin-like (UbLs) modifications such as ISGylation, Neddylation, UFMylation, SUMOylation (3, 4). These modifications virtually control all aspects of innate immune signaling (5, 6). A concerted series of enzymatic processes leads to the covalent attachment of Ub/UbLs to mainly lysine residue of substrate proteins. It involves an activation enzyme (E1), a conjugation enzyme (E2), and ligating enzyme (E3). E3-Ub ligases recognize the substrate simultaneously interacts with the Ub-conjugated E2 and the substrate protein and mediates the isopeptide bond formation between the Ub and a lysine residue of the substrate protein (7). Modification of a signaling protein by Ub or UbLs eventually impacts its functional activity and/or stability.

More than 600 human E3-ligases have been identified so far, among them are members of the tripartite motif (TRIM) family that are encoded within the MHC-I locus of the human DNA (8). The review by Jia et al. highlights the role of TRIMs in regulating innate immunity that impacts infections or auto-inflammatory conditions. Beyond classical ubiquitination, TRIM proteins like TRIM38 mediate SUMOylation in RNA-sensing (RIG-I/MDA5) and DNA-sensing (cGAS/STING) pathways. A role of TRIM7 in the negative regulation of innate immune response upon RNA-virus infection was revealed by the study of Yang et al. TRIM7, also known as E3 ubiquitin-protein ligases RING-finger protein 90 (RNF90) promotes K48-linked ubiquitination of the important RNA-sensing signaling adaptor MAVS. This eventually leads to the proteasomal degradation of MAVS and consequently decreased innate immune signaling in response to RNA viruses. Of note, the negative regulation of MAVS by TRIM7 may protect against aberrant spontaneous activation of immune responses under physiological conditions.

Ubiquitin has seven lysine residues (Lys6, Lys11, Lys27, Lys29, Lys33, Lys48, and Lys63). The mode of Ub lysine-lysine interconnection determines the fate of the substrate protein (9). In addition to lysine-linked chains, linear chains can be formed that are linked *via* the N-terminal first methionine. This is mediated by the specialized E3-ligase complex Linear ubiquitin chain assembly complex (LUBAC) (10, 11). The study by Miyashita et al. shows that the autophagy cargo, Nuclear dot protein 52 kDa (NDP52, also known as calcium binding and coiled-coil domain 2, CALCOCO2) interacts with the LUBAC complex *via* the HOIP subunit. Although this interaction did not affect the E3-ligase activity of the LUBAC, NDP52 negatively regulates TNF-α secretion, antiviral signaling, and apoptosis, as well as xenophagosome formation.

Despite forming a covalent bond between the substrate and ubiquitin chain, modifications by ubiquitination are reversible. Homeostasis is maintained by a balance between PTM formation by E3-ligases and the removal of ubiquitin chains by deubiquitinases (DUBs) (12). Currently, more than 100 human DUBs have been identified, and the role of major DUBs in type-I IFN signaling during viral infection was comprehensively reviewed by Qian et al. In addition, it was highlighted that the DUBs are not always required to be catalytically active to regulate signal transduction. DUBs can also have specific antiviral activity, as shown by Gao et al. Specifically, the DUB ubiquitin-specific protease 8 (USP8) may suppress HIV-1 infectivity by counteracting Vif-induced APOBEC3G degradation. Their work also revealed other DUBs like USP7, USP33, and USP37 that play a role as host-encoded restriction factors to prevent degradation facilitated by HIV-1 accessory proteins Vpr, Vpu, and Vpx.

However, viruses have evolved strategies to interfere with the ubiquitin system to counteract the host defenses and promote their replication. To this end, viruses encode proteins that mimic key components of Ub and UbL ligases or deubiquitinates (13, 14). For example, poxviruses encode E3-ubiquitin ligase and adaptor proteins belonging to PRANC, ANK/BC, BBK, P28/RING, and MARCH proteins. Cui et al. reviewed the role of these five categories of proteins in host immune evasion. Accessory proteins of HIV, such as Nef exploit ubiquitin regulatory factors to dampen innate signaling. Castro-Gonzalez et al. provide a perspective on Nef’s impact on autophagy and apoptotic pathways triggered by Nef-promoted mono-ubiquitination of BCL-2 by PRKN. Mono-ubiquitination enhances the interaction of BCL-2 with BECN1, thereby diminishing autophagy initiation (15).

While ubiquitination is undoubtedly among the most studied PTMs, UbL modifications like ISGylation, SUMOylation, and Neddylation etc. have recently gained increased attention. In principle, similar cascades as classical ubiquitination are required to mediate conjugation to substrates. In their review, Thery et al. discussed different mass-spectrometry-based approaches to identify the substrates of Interferon-stimulated gene 15 (ISG15) and ISGylation sites. Mohanty et al. studied interaction dynamics between NEDD8 and E3-ligase Culling-RING ligases (CRLs). All-atom MD simulations combined with NMR spectroscopy revealed the interactions that stabilize the open and closed conformation of NEDDylated-Cul5^CTD^. In addition, they define the role of NEDD8 in the catalytic confirmation of Cullin-Rbx1 and the effect of NEDD8 deamidation on CRL activity.

Although a significant focus of the Research Topic is Ub and UbL proteins in the human system, we did not restrict ourselves to one species only. In shrimps, Wang et al. identify a viral serine/threonine protein kinase WSV083 that phosphorylates the nuclear transcription factor Tcf, triggering its ubiquitination and proteasome mediated degradation. Thus, the antiviral function of Tcf is reduced. Shephard et al. studied the structure of chicken 2’-5’ oligoadenylate synthetase-like proteins (OASL), a key enzyme in innate immunity, whose UbL tandem domain shares similarity to human ISG15 and, unlike mammalian OASL, it can be conjugated to proteins. This led them to speculate that OASL may have a similar function as ISG15 in the absence of the latter.

In this collection we have assembled novel research and overview articles covering different stages of the ubiquitination and deubiquitination cascades, non-ubiquitin modifiers, and extending our understanding of the role of Ub and UbLs in innate immune regulation of non-human species. State-of-the-art proteomics-based and NMR spectroscopy-based technologies that could be applied to study interactions and structural conformations were presented. Although our collection has certainly expanded our knowledge about the role of Ub and UbL modifications in antiviral innate immune responses, there are still a lot of open questions. Are specific ubiquitin chains more prevalent for one or another innate immune pathway? Are the known conjugated proteins the only UbL proteins? How important are the mixed linkage-type chains in innate immunity? Can the proteolysis-targeting chimera (PROTAC) technology be used in the therapeutic regulation of innate immune response? Thus, we are also looking forward to exciting future work on Ub and UbL modifiers as the major regulatory PTMs in innate immunity.

## Author contributions

All the authors equally contributed in drafting, reviewing, and editing the manuscript critically for intellectual components and approved it for final submission. Original draft by SG.
